# The Care of the Children during a Hot Summer

**Published:** 1908-09

**Authors:** J. Bernard Dawson

**Affiliations:** Late House Surgeon, The Children s Hospital, Brighton


### PROGRESS IN MEDICAL SCIENCE.

---

### The Care of the Children During a Hot Summer.

BY J. BERNARD DAWSON, M. B., F.R.C.S.
Late House Surgeon, The Children s Hospital, Brighton.

(*The Hospital.*)

TO every medical practitioner, whether he be engaged in private work or in that of an institution, there falls a share of minor infantile ills attributable to the atmospheric conditions which prevail during hot weather. One may feel competent to manage a case of acute pneumonia or intussusception and yet be at a loss when confronted with a vague maternal description of the sickness of a child. To parents, however, these ills are very real, and much discredit may come to a doctor who fails to relieve or satisfy.

Setting aside the large subject of the symptoms, diagnosis, and treatment of acute summer diarrhea, there are certain lines of inquiry and treatment to be adopted when face to face with the lesser evils attendant upon the juvenile population at this season of the year.

Unfortunately, the conditions of life among the poorer classes in large towns are such that little can be done at present to alleviate the suffering of the children. The apathy of parents, the regulations of schools, and the denseness of population handicap the profession too severely for adequate or practical handling of this problem. Something, however, can be done, even among the poorest, if regular, reiterated, and importunate attention be drawn to the benefits to be derived from sterilisation of milk, destruction of waste vegetable matter, avoidance of doubtful fruit, fish, and ice cream, and above all to an intelligent use of the green oases with which our big cities are scattered. Among the better-to-do and educated classes much can be done to prevent and to cure the troubles attendant upon high temperature.

1. *Clothing.*—During the past ten years great revolutions have taken place in the clothing of children. Fortunately these changes have been, in the main, directed by logical ideas of hygiene and health. Nevertheless there has been, as in all reaction, too long a swing of the pendulum. The bare or sandal-footed infant, clad in scanty garments of cotton and scantier underclothing, is undoubtedly pleasing to the asthetic eye, but it is by no means certain that such attire is ideal from the standpoint of health. Too often the clothing is cut down to a summer standard at the expense of underclothing, leaving much of the lower limbs and pelvis exposed. Two evils are attendant upon this: (1) The prevalence of dust, the increased outdoor life, the delights of the hayfield or the orchard make it unwise that half of a child's body should be exposed to the attacks of insects and bacteria which abound in such surroundings; (2) even in our best English summers, the advent of evening brings with it a marked lowering of the temperature and heavy dews. Much evil can be wrought by the exposure of underclad, over-heated children, tired at the end of a day's play, to this change of atmosphere. The summer clothing of children should by all means be as light and airy as possible, but it should also aim at an adequate protection of legs and abdomen from the ravages of mosquitoes, midges, harvest-bugs, hay-spiders, and insect plagues in general.

2. *The Night Nursery.*—Little need be said on this subject, for the value of the open window, the light bed-hangings, and severe furniture now fashionable is apparent to all thinking parents. But in the midst of a hot month to leave a window wide open without preventing the entry of parasitic insects is merely to ask for restless children, broken sleep, and matutinal peevishness. No Indian ayah would leave her charges unprotected from the pests of an Eastern night. Those who have had the misfortune to treat the results of their onslaughts know what a real pest the English mosquito has become. Muslin stretched upon a frame made to fit the window affords a simple and inexpensive means of admitting air freely, but excluding the unwelcome guests.

3. *Diet.*—There is little doubt that vegetarianism is for children the safest and most salutary mode of life during hot weather. The inclusion of light soups and small quantities of meat, while doing little or no harm, seems unnecessary at a season when fresh fruit and vegetables are so abundant. Probably the citrates, malates, and tartrates of such fresh vegetation exert a corrective influence upon the intestinal aud hematic functions. Eggs are a most useful addition, and their substitution for the traditional breakfast bacon is most beneficial.

Porridge, popularly supposed to be "heating," is better avoided. As a bulky carbohydrate food it is unnecessary at a season when meals should be light and undue fermentation is easily established. Fish, if above suspicion and lightly boiled—not fried—is an admirable addition to the juvenile dietary. Sweets, chocolate, ices, even when of the highest character, should be treated with reserve and given sparingly.

4. *Exercise and Recreation.*—A mistake often made is to send children out when the heat is extreme. Outdoor exercise cannot be too highly praised, but exposure of children to a July sun at midday is injurious and ofttimes cruel. The shady spots of such happy places as Kensington Gardens are limited, and many children can be seen there daily, obviously suffering from heat, and heat alone. Unless deep shade can be assured children are happier and healthier in a well-aired nursery than out of doors during the hottest parts of the day. Before 10 a.m. and after 5 p.m. they may be out for games to their hearts' content, but between those hours great care and discretion should be exercised where little children are concerned.

5. *Medicine.*—Observance of simple hygienic rules should render unnecessary the resort to drugs. Nevertheless there are occasions when such are useful. The addition of a little oil of eucalyptus or oil of bergamot to the morning bath will help in no small way to ward off the attentions of insects. For stings and bites nothing is better than:

℞ Liquoris carbonis detergentis ........................ ʒj.

Aquam ........................................ ad. ℥x

Misce fiat lotio.

For slight irregularities of defecation the old-fashioned succus taraxaci or fractional doses of calomel are usually sufficient.

For papular eruptions attributable to excessive heat, pulvis sodæ tartaratæ effervescens, given as follows, will be found very useful:

℞ (A) Sodæ tartaratæ ............................ gr. xx.
Sodii bicarbonatis ........................ gr. v.
Misce fiat pulv.
(B) Acidi tartarici ............................ gr v
Fiat pulv.

S. Dissolve A in a wineglassful of cold water, then add powder B and give whilst effervescing.

The regular use of the popular lemon-kali has a similar effect.

6. *School*—In those classes of society whose children are not under the hard and fast rules of compulsory attendance at school, useful latitude can be allowed in attendance during the hot months. Regular but light scholastic work occupying the middle of the day is perhaps the best way of occupying a child's time, provided that the conditions are good. It would be well if all parents would pay a visit to the room their children occupy for their lessons during a hot morning. The overcrowding of children into an imperfectly or improperly ventilated room demands a heavy toll on their health and energy.

The introduction of State open-air schools sets an example to the wealthier classes. Children enjoy their work, gain more benefit therefrom, and do no injury to their health, if lessons are given in a suitably chosen and shaded spot in garden or field.

7. *Holidays*.—From a child's standpoint the choice of a holiday ground is of great importance. To take a child from Hampstead to a relaxing South-Coast watering-place does more harm than good. Similarly, to remove from a school in a bracing north county to a Kentish holiday haunt is an injustice to health and common sense. Big seaside towns, where the air is often a pot-pourri of asphalt, petrol, and dust, are again often of no benefit.

Many villages high up on the South Downs, and many glorious seaside villages in the south-west counties of England and Wales, offer ideal air and surroundings for children, albeit the band be absent and the golf links inferior.

---

In cases of relapsing epididymitis the prostate is often the source of infection even when distinct signs of chronic urethritis are absent.—*International Jour. Surg.*

